# *Arabidopsis* and rice showed a distinct pattern in ZIPs genes expression profile in response to Cd stress

**DOI:** 10.1186/s40529-018-0238-6

**Published:** 2018-09-25

**Authors:** Xin Zheng, Liang Chen, Xiaofang Li

**Affiliations:** 1 Key Laboratory for Agricultural Water Resources, Center for Agricultural Resources Research, Institute of Genetics and Developmental Biology, Chinese Academy of Sciences, Shijiazhuang, 050021 Hebei People’s Republic of China; 2 CMLR, Sustainable Minerals Institute, The University of Queensland, Brisbane, QLD 4072 Australia

**Keywords:** ZIP family, Cd, Metal cation transporter, Gene expression, Cd uptake

## Abstract

**Background:**

Plant ZIP genes represent an important transporter family involved in metal transport. Evidence has implied that some ZIPs may contribute to plant Cd uptake, but a genome-wide examination of ZIPs’ role in Cd tolerance and uptake has rarely been reported. In this study, a genome-wide bioinformatic screening of candidate ZIP genes in *Arabidopsis* and rice was performed, followed by a systematic determination of their expression profile in response to Cd stress. Typical up-regulated ZIPs genes were then expressed in yeast cells to examine their effect on hosts’ Cd uptake.

**Results:**

A total of 27 ZIP genes in *Arabidopsis* and rice were screened out based on sequence similarity. In *Arabidopsis*, Cd exposure strongly impacted the expression of most ZIPs, among which *AtIRT1*, *AtIRT2*, *AtIRT4 AtZIP9*, *AtZIP10* and *AtZIP12* were sharply up-regulated and *AtIRT3*, *AtIRT5* were significantly down-regulated in root. In rice, all tested genes in shoot except for *OsIRT1* and *OsIRT12* were sharply up-regulated, while *OsIRT1* and *OsZIP1* in root were significantly down-regulated. Interestingly, some genes like *AtIRT3*, *AtZIP5*, *AtZIP12*, *OsIRT1* and *OsZIP1* showed converse expression regulation when subject to the tested Cd stress. When expressed in yeast cells, three ZIPs, *AtIRT1*, *OsZIP1* and *OsZIP3*, caused a substantial increase in Cd sensitivity and Cd accumulation of the host cells.

**Conclusions:**

In conclusion, this study revealed a distinct pattern in ZIPs family genes expression between *Arabidopsis* and rice in response to Cd stress. *Arabidopsis* mainly up-regulated root ZIPs genes, while rice mainly up-regulated shoot ZIPs genes. Three genes, *AtIRT1*, *OsZIP1* and *OsZIP3*, conferred an increased Cd accumulation and sensitivity to Cd stress when expressed in yeast cells, further implying their roles in Cd uptake in plants.

**Electronic supplementary material:**

The online version of this article (10.1186/s40529-018-0238-6) contains supplementary material, which is available to authorized users.

## Background

The zinc(Zn)-regulated/iron(Fe)-regulated transporter-like family proteins (ZIPs) are membrane-located proteins for cations transport (Eng et al. 1998; Guerinot 2000). They have been found to exist broadly in prokaryotic cells, fungi, plants and mammalians. In plants, ZIPs have been identified in both dicots and monocots, such as *Arabidopsis* (Grotz et al. 1998; Milner et al. 2013), rice (Chen et al. 2008), maize (Li et al. 2013), medicago (Lopez-Millan et al. 2004; Stephens et al. 2011) and barely (Tiong et al. 2015). Grotz et al. identified five ZIP genes (*IRT1*, *ZIP1*-*4*) in *Arabidopsis* (Grotz et al. 1998), and later up to 11 ZIP genes from *Arabidopsis* were detected bioinformatically (Guerinot 2000). Roles of *ZIP1*-*12* from *Arabidopsis* in Zn transport were explored experimentally (Milner et al. 2013). More recently, 18 ZIPs from *Arabidopsis* and 16 ZIPs from rice were annotated (Ivanov and Bauer 2017).

In *Arabidopsis* and rice, only a small number of ZIPs have been examined for biological functions in plant till now. *Arabidopsis IRT1* is a well-studied ZIP gene first identified as a crucial transporter for plant Fe uptake (Varotto et al. 2002; Vert et al. 2002). *Arabidopsis IRT1* can be induced by iron deficiency (Korshunova et al. 1999; Connolly et al. 2002), and may play a role in Mn/Zn transport as well (Korshunova et al. 1999; Rogers et al. 2000; Connolly et al. 2002). Biological functions in Zn/Fe transport of *AtIRT2* (Vert et al. 2001, 2009), *AtZIP1/2* (Grotz et al. 1998; Wintz et al. 2003; Milner et al. 2013), *OsIRT1* (Nakanishi et al. 2006; Lee and An 2009; Ishimaru et al. 2006; Bughio et al. 2002) and *OsZIP4/5/8* (Ishimaru et al. 2005; Chen et al. 2008; Lee et al. 2010a, b; Yang et al. 2009) have also been examined in the past decade.

A few studies have also implied that ZIPs may be involved in Cd transport. Yeast cells expressing *AtIRT1* showed increased Cd sensitivity (Rogers et al. 2000; Vert et al. 2001), and IRT1-dependent Fe/Mn/Zn uptake was inhibited by excess Cd (Eide et al. 1996; Korshunova et al. 1999). The *Arabidopsis IRT1* knock-out mutant *irt1*-*1* exhibited reduced Cd sensitivity and Cd accumulation (Vert et al. 2002; Fan et al. 2014), while overexpression of *AtIRT1* increased Cd sensitivity in *Arabidopsis* (Connolly et al. 2002). *AtIRT2*, phylogenetically similar to *AtIRT1*, increased Cd uptake when overexpressed in *Arabidopsis* (Vert et al. 2009), though the yeast cells expressing *AtIRT2* exhibited no altered Cd sensitivity (Vert et al. 2001). In rice, expression of *OsIRT1* and *OsIRT2* made the cells more sensitive to Cd and increased Cd accumulation (Nakanishi et al. 2006; Lee and An 2009). Nonetheless, we still know little about the roles of most of the ZIPs genes in Cd stress response in *Arabidopsis* and rice.

In this study, genome-wide ZIPs identification in *Arabidopsis* and rice was performed with rigorous evolutional analysis. A comparative examination of genome-wide expression profile of ZIPs in *Arabidopsis* and rice in response to Cd stress were carried out. Their role in Cd uptake of typical ZIPs responding to Cd stress was further tested by expressing them in yeast. As expected, most identified ZIPs gene expression responded remarkably to Cd stress, while unexpectedly it was found that *Arabidopsis* and rice showed a distinct pattern in ZIPs genes expression profile. These results may help to elucidate the plants’ genetic basis for Cd translocation via a ZIPs-dependent pathway.

## Materials and methods

### Bioinformatics

Genomic query of *Arabidopsis* and rice ZIP family genes was performed online using the PLAZA database (http://bioinformatics.psb.ugent.be/plaza/). The sequences of 27 ZIP genes were retrieved manually from the TAIR database (http://www.arabidopsis.org/index.jsp) and the TIGR database (http://rice.plantbiology.msu.edu/index.shtml).

TM regions and other domains of the identified ZIPs gens were predicted through the TMHMM Server (http://www.cbs.dtu.dk/services/TMHMM-2.0/) and UniProtKB database (http://www.uniprot.org/), following a routine procedure.

### Experimental design

*Arabidopsis thaliana* ecotype Col-0 and *Oryza sativa ssp. japonica* (*cv.* Taichung65) were subject to Cd inhibition test. For *Arabidopsis*, plants were germinated on Murashige and Skoog (MS; pH 5.7) solid medium containing 1% (w/v) sucrose. A total of 60 1-week-old seedlings were transferred to MS (control) or MS with 300 μM CdCl_2_ (Cd stress treatment) solid medium, and grown for 3 days in a controlled chamber environment under a 16/8 h photoperiod at 22 °C. For rice, seedlings were germinated hydroponically in distilled water. A total of six 10-day-old seedlings were then subject to a hydroponic culture in distilled water (control) or 300 μM CdCl_2_ solution (Cd stress treatment) for 3 days under 16/8 h photoperiod at 25 °C. The Cd concentration used in this study was selected based on our pilot experiment.

After Cd stress treatment, the shoot and root tissues were harvested and frozen immediately in liquid nitrogen. Total RNA was isolated from the tissues using Trizol reagent (Invitrogen, Corp., Carlsbad, CA, USA) and treated with DNase I (Promega, Madison, WI, USA). A total of 5 μg RNA was used for reverse transcription with PrimeScript™ RT reagent Kit (Takara Biotechnology Co. Ltd., Dalian, China) following the manufacturer’s protocol.

Quantitative Real-Time PCR (qPCR) was performed in a Bio-Rad CFX Connect™ Real-Time PCR Detection System (Hercules, CA, USA) using a SYBR Green Premix Ex Taq (Takara). The PCR parameters were set as: 95  °C for 5 min, followed by 40 cycles of 95  °C for 10 s and 60  °C for 30 s. *Arabidopsis ACTIN* gene (GenBank accession number NM_179953) and rice *ACTIN* gene (GenBank accession number XM_015774830) were used as internal references. Relative gene expression levels were detected using the 2^−ΔΔCT^ method (Livak and Schmittgen 2001). Gene expression level was normalized using shoot expression level of each gene in the controls as a calibrator. All primer sequences are listed in the Additional file 1: Table S1.

Cd sensitivity analysis was performed using drop assay. Full-length coding sequence (CDS) was obtained via PCR amplification (see primers in Additional file 1: Table S2), and ligated into pCEV-G1-Km vector under the *PGK1* promoter. The recombinant plasmids were then introduced into *Saccharomyces cerevisiae* (strain AH109) using a lithium acetate-based method. Transformed cells were cultured in Yeast Extract Peptone Dextrose (YPD) media with 300 μg/mL geneticin (G418), harvested by centrifugation, and resuspended in water (OD_600_ = 1.0), followed by a serial dilution. A total of 5 μL of each dilution was inoculated onto the YPD plates containing 300 μg/mL G418 and 50 μM CdCl_2_. Cells harbouring empty pCEV-G1-Km were used as a negative control. The plates were incubated at 28 °C for 5 days and the growth of the colonies was subsequently observed.

For the determination of Cd concentration in transformed yeast cells, cells expressing *ZIPs* were harvested after 12 h with 50 μM CdCl_2_ treatment. Cd was determined using a flame atomic absorption spectrometry (F-AAS) quantitative method. In Brief, cells in the liquid culture were harvested by centrifugation at 4000×*g* and washed three times with 3% NaCl solution. The cells were then oven-dried, weighed and digested using 4 mL 65% HNO_3_. The digested mixture was dissolved in 3 mL Millipore^®^ water and subject to Cd determination using a Zeenit 700 P Atomic Absorption Spectrometer (Analytik Jena, Germany) equipped with a flame atomizer. CRM Laver (GWB10023, certified by IGGE) was used as a standard reference material for Cd determination.

### Data analysis

Phylogenetic analysis was performed using MEGA 7 (Kumar et al. 2016). The model of ZIP gene structure was constructed using Gene Structure Display Server (http://gsds.cbi.pku.edu.cn/).

Statistical analysis was performed using SPSS 21.0 (IBM, New York, USA). Unpaired two-tailed t test was performed for comparison between the controls and the Cd stress group.

## Results and discussion

**Fig. 1:**
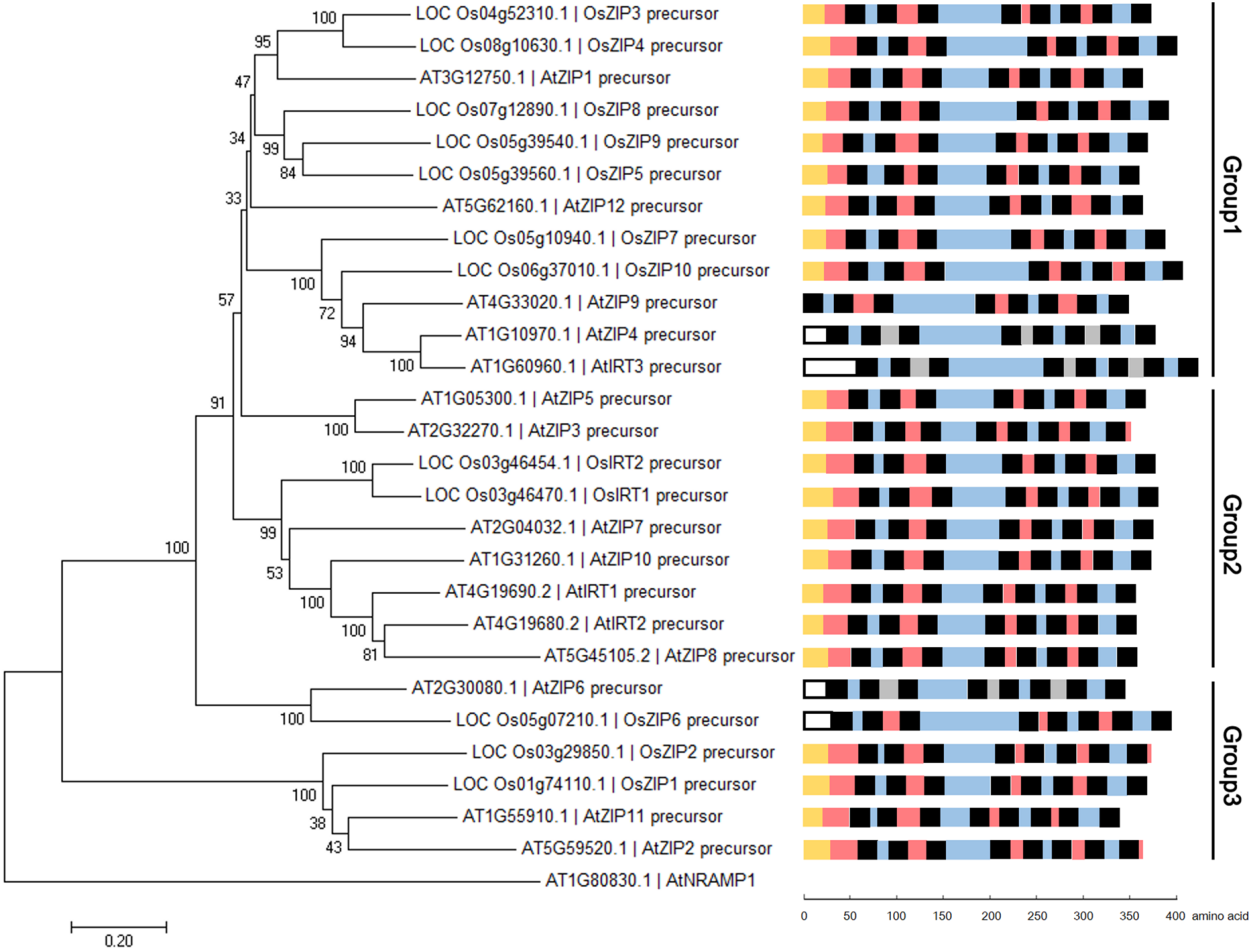
Phylogenetic relationship of identified ZIPs in *Arabidopsis* and rice. The Neighbor-Joining tree was generated using MEGA7 with 1000 bootstrap replicates, and rooted to the AtNRAMP1. Topological structure was predicted using UniProtKB and TMHMM. Black boxes indicate TM regions. Pink boxes indicate extracellular regions. Blue boxes represent cytoplasmic regions. Gray boxes represent luminal regions. Yellow boxes represent SP region. Uncharacteristic SPs are represented by the white boxes

In this study, 15 candidate ZIP genes from *Arabidopsis* and 12 from rice were screened out based on sequence similarity. The number of ZIPs identified here was similar to previous studies (Ivanov and Bauer 2017; Guerinot 2000). Evolutionary analysis further indicated that all of these ZIP genes contain 1–3 introns (Additional file 1: Figures S1 and S2), whose protein precursors comprise eight TM regions (~ 20 aa length), one variable region with a conserved HG repeat and a typical signal peptide (SP) located on the N-terminal (Fig. 1). AtZIP13 and OsZIP13, which were previously annotated as putative Zn transporter (Ivanov and Bauer 2017), contain more TM domains. *AtZTP29*, *AtIAR1*, *OsIAR1*, *OsZIP11* and *OsZIP12* contain more than 10 exons. These ZIP-like genes seem to be phylogenetically distant from *SpZRT1* and *AtIRT1* and were not tested in this study. Phylogenetic clustering of the tested 27 ZIPs identified three subgroups, which is similar to previous study (Ivanov and Bauer 2017), namely the seed plant-specific group, the mixed plant group, and the mixed group1/2 (Fig. 1).

**Fig. 2:**
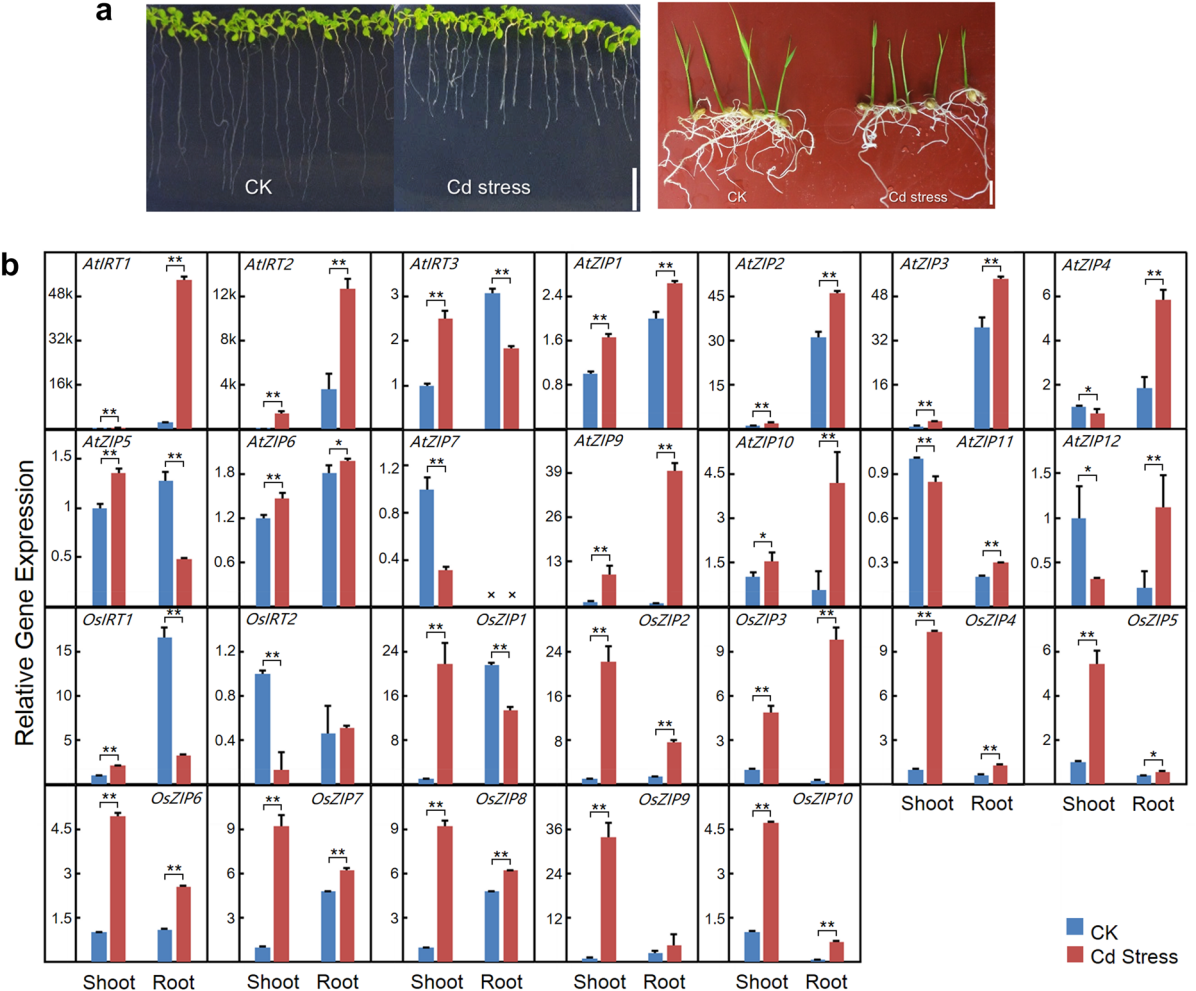
Expression profiles of ZIP genes of *Arabidopsis* and rice in response to Cd stress. **a** 1-week-old and 10-day-old seedlings of *Arabidopsis* and rice were treated with 300 μM CdCl_2_ for 3 days. Scale bars indicate 1 cm. **b** Changes in the expression of the 26 ZIP genes in response to Cd exposure Gene expression level was normalized using shoot expression level of each gene in the controls as the calibrator. (n = 3, Student *t* test, *indicates P < 0.05, **indicates P < 0.01)

Most previous studies on ZIPs’ biological functions focused on Zn/Fe/Mn/Cu uptake in yeast cells (Table 1), and expression profile of most ZIPs (except for *AtIRT1/2* and *OsIRT1/2*) in response to Cd remained unknown. In this study, the genome-wide expression profile of ZIP genes in response to Cd stress in *Arabidopsis* and rice were quantified using qPCR. To induce a substantial stress response, 300 μM Cd in culture medium was applied based on our pilot experiment. The 3 days’ treatment obviously inhibited seedling growth and root elongation of both *Arabidopsis* and rice, and rice seedling height was also reduced (Fig. 2a). It was reported that even moderate Cd exposure can cause toxic symptoms and increased Cd accumulation in *Arabidopsis* (Fan et al. 2014) and rice (Rafiq et al. 2014). The Cd level used here significantly reduced the root length and seedling dry weight (Additional file 1: Figure S3), and was thus supposed to induce rapid expressional changes in the tested plants.

**Table 1:** Locations and known functions of ZIP proteins in *Arabidopsis* and rice

Gene name	Locus	Complementation of yeast metal uptake mutants (y/n)	Validated location in plant	Experimental evidence for potential function in Cd uptake	References
*AtIRT1*	At4g19690	∆*fet3*∆*fet4* (Y); ∆*zrt1*∆*zrt2* (Y); ∆*ctr1* (N); ∆*smf1* (Y)	Early endosome, vacuole, trans-Golgi network and cell membrane; root epidermis, flower	Increased Cd sensitivity of overexpression plant/yeast Reduced Cd sensitivity of *irt1* Inhibited IRT1-dependent Fe/Mn/Zn uptake by Cd in yeast Reduced Cd uptake of *irt1*	Eide et al. (1996), Vert et al. (2002, 2001), Korshunova et al. (1999), Rogers et al. (2000), Connolly et al. (2002), Varotto et al. (2002), Henriques et al. (2002), Nishida et al. (2011), Shin et al. (2013), Potocki et al. (2013), Fan et al. (2014), Barberon et al. (2014), Blum et al. (2014)
*AtIRT2*	At4g19680	∆*fet3*∆*fet4* (Y); ∆*zrt1*∆*zrt2* (Y); ∆*smf1* (N)	Intracellular vesicles; root epidermis	No altered Cd sensitivity of overexpression yeast Increased Cd uptake and *IRT1* expression of overexpression plant	Vert et al. (2001, 2009), Wintz et al. (2003), Varotto et al. (2002)
*AtIRT3*	At1g60960	∆*Spzrt1* (Y); ∆*zrt1*∆*zrt2* (Y); ∆*fet3*∆*fet4* (Y); ∆*smf1* (N)	Cell membrane; broadly expressed	No altered Cd sensitivity of overexpression yeast	Lin et al. (2009), Talke et al. (2006), Shanmugam et al. (2011), Hammes et al. (2005)
*AtZIP1*	At3g12750	∆*zrt1*∆*zrt2* (Y); ∆*fet3*∆*fet4* (N); ∆*ctr1*∆*ctr3* (N); ∆*smf1* (Y)	Vacuolar; predominantly root stele and leaf vasculature	Inhibited ZIP1-dependent Zn uptake by Cd in yeast, to a less extent	Grotz et al. (1998), Milner et al. (2013)
*AtZIP2*	At5g59520	∆*zrt1*∆*zrt2* (Y); ∆*fet3*∆*fet4* (N); ∆*ctr1*∆*ctr3* (N); ∆*smf1* (Y); ∆*ctr1* (Y)	Cell membrane; predominantly mature root stele	Inhibited ZIP2-dependent Zn uptake by Cd in yeast	Grotz et al. (1998), Milner et al. (2013), Wintz et al. (2003)
*AtZIP3*	At2g32270	∆*zrt1*∆*zrt2* (Y); ∆*fet3*∆*fet4* (N); ∆*ctr1*∆*ctr3* (N); ∆*smf1* (N)	Predominantly root	Inhibited ZIP3-dependent Zn uptake by Cd in yeast, to a less extent	Grotz et al. (1998), Talke et al. (2006), Milner et al. (2013)
*AtZIP4*	At1g10970	∆*zrt1*∆*zrt2* (N); ∆*fet3*∆*fet4* (N); ∆*ctr1* (Y)	Root and leaf	N/A	Grotz et al. (1998), Talke et al. (2006), Wintz et al. (2003)
*AtZIP5*	At1g05300	∆*zrt1*∆*zrt2* (N); ∆*fet3*∆*fet4* (N); ∆*ctr1*∆*ctr3* (N); ∆*smf1* (Y)	Root	N/A	Milner et al. (2013), Wintz et al. (2003)
*AtZIP6*	At2g30080	∆*zrt1*∆*zrt2* (N); ∆*fet3*∆*fet4* (N); ∆*ctr1*∆*ctr3* (N); ∆*smf1* (Y)	Root	N/A	Milner et al. (2013), Hammes et al. (2005)
*AtZIP7*	At2g04032	∆*zrt1*∆*zrt2* (Y); ∆*fet3*∆*fet4* (Y); ∆*ctr1*∆*ctr3* (N); ∆*smf1* (Y)	N/A	N/A	Milner et al. (2013)
*AtZIP8*	At5g45105	∆*zrt1*∆*zrt2* (N); ∆*fet3*∆*fet4* (N); ∆*ctr1*∆*ctr3* (N); ∆*smf1* (N)	N/A	N/A	Milner et al. (2013)
*AtZIP9*	At4g33020	∆*zrt1*∆*zrt2* (N); ∆*fet3*∆*fet4* (N); ∆*ctr1*∆*ctr3* (N); ∆*smf1* (Y)	Root and shoot	N/A	Talke et al. (2006), Milner et al. (2013), Wintz et al. (2003), Inaba et al. (2015)
*AtZIP10*	At1g31260	∆*zrt1*∆*zrt2* (Y); ∆*fet3*∆*fet4* (N); ∆*ctr1*∆*ctr3* (N); ∆*smf1* (N)	N/A	N/A	Milner et al. (2013)
*AtZIP11*	At1g55910	∆*zrt1*∆*zrt2* (Y); ∆*fet3*∆*fet4* (N); ∆*ctr1*∆*ctr3* (N); ∆*smf1* (N)	N/A	N/A	Milner et al. (2013)
*AtZIP12*	At5g62160	∆*zrt1*∆*zrt2* (Y); ∆*fet3*∆*fet4* (N); ∆*ctr1*∆*ctr3* (N); ∆*smf1* (N)	Root	N/A	Milner et al. (2013), Inaba et al. (2015)
*OsIRT1*	LOC_Os03g46470	∆*fet3*∆*fet4* (Y); ∆*ctr1* (N); ∆*zrt1*∆*zrt2* (N); ∆*smf1* (N); ∆*frt1*∆*fet4*∆*fre1*(Y); ∆*frt1*∆*fet1*∆*fre3*(Y)	Cell membrane; mainly in root epidermis (the inner layer of the cortex, and the stele) and stems (companion cells)	Increased Cd sensitivity of overexpression plant Increased Cd sensitivity and Cd uptake of overexpression yeast	Ishimaru et al. (2006), Bughio et al. (2002), Lee and An (2009), Nakanishi et al. (2006), Ishimaru et al. (2005)
*OsIRT2*	LOC_Os03g46454	∆*fet3*∆*fet4* (Y); ∆*ctr1* (N); ∆*zrt1*∆*zrt2* (N); ∆*smf1* (N)	Cell membrane	Increased Cd sensitivity and Cd uptake of overexpression yeast	Ishimaru et al. (2006), Nakanishi et al. (2006)
*OsZIP1*	LOC_Os01g74110	∆*zrt1*∆*zrt2* (Y); ∆*smf1* (Y); ∆*fet3*∆*fet4* (N)	Broadly expressed	Increased Cd sensitivity of overexpression yeast Inhibited ZIP1-dependent Zn uptake by Cd in yeast	Ramesh et al. (2003, Ishimaru et al. (2005), Chen et al. (2008)
*OsZIP2*	LOC_Os03g29850	∆*zrt1*∆*zrt2* (N);	N/A	No altered Cd sensitivity of overexpression yeast	Ramesh et al. (2003)
*OsZIP3*	LOC_Os04g52310	∆*zrt1*∆*zrt2* (Y); ∆*smf1* (Y); ∆*fet3*∆*fet4* (N)	Mainly induced by zinc deficiency to higher levels in roots	No altered Cd sensitivity of overexpression yeast Mildly increased ZIP3-dependent Zn uptake by Cd in yeast	Ramesh et al. (2003, Ishimaru et al. (2005), Chen et al. (2008)
*OsZIP4*	LOC_Os08g10630	∆*zrt1*∆*zrt2* (Y); ∆*frt1*∆*fet1*∆*fre3*(N)	Cell membrane; phloem cells of leaves, roots and meristem	N/A	Ishimaru et al. (2005), Chen et al. (2008)
*OsZIP5*	LOC_Os05g39560	∆*zrt1*∆*zrt2* (Y)	Cell membrane; mainly panicle	N/A	Chen et al. (2008), Lee et al. (2010a)
*OsZIP6*	LOC_Os05g07210	N/A	Root, shoot and panicle	Increased Cd uptake of overexpression cells	Chen et al. (2008)
*OsZIP7*	LOC_Os05g10940	N/A	Root, shoot and panicle	N/A	Chen et al. (2008), Yang et al. (2009)
*OsZIP8*	LOC_Os07g12890	N/A	Cell membrane; mainly root and panicle	N/A	Chen et al. (2008), Lee et al. (2010b), Yang et al. (2009)
*OsZIP9*	LOC_Os05g39540	N/A	Root, shoot and panicle	N/A	Chen et al. (2008)
*OsZIP10*	LOC_Os06g37010	N/A	N/A	N/A	N/A

N/A represents not available

**Fig. 3:**
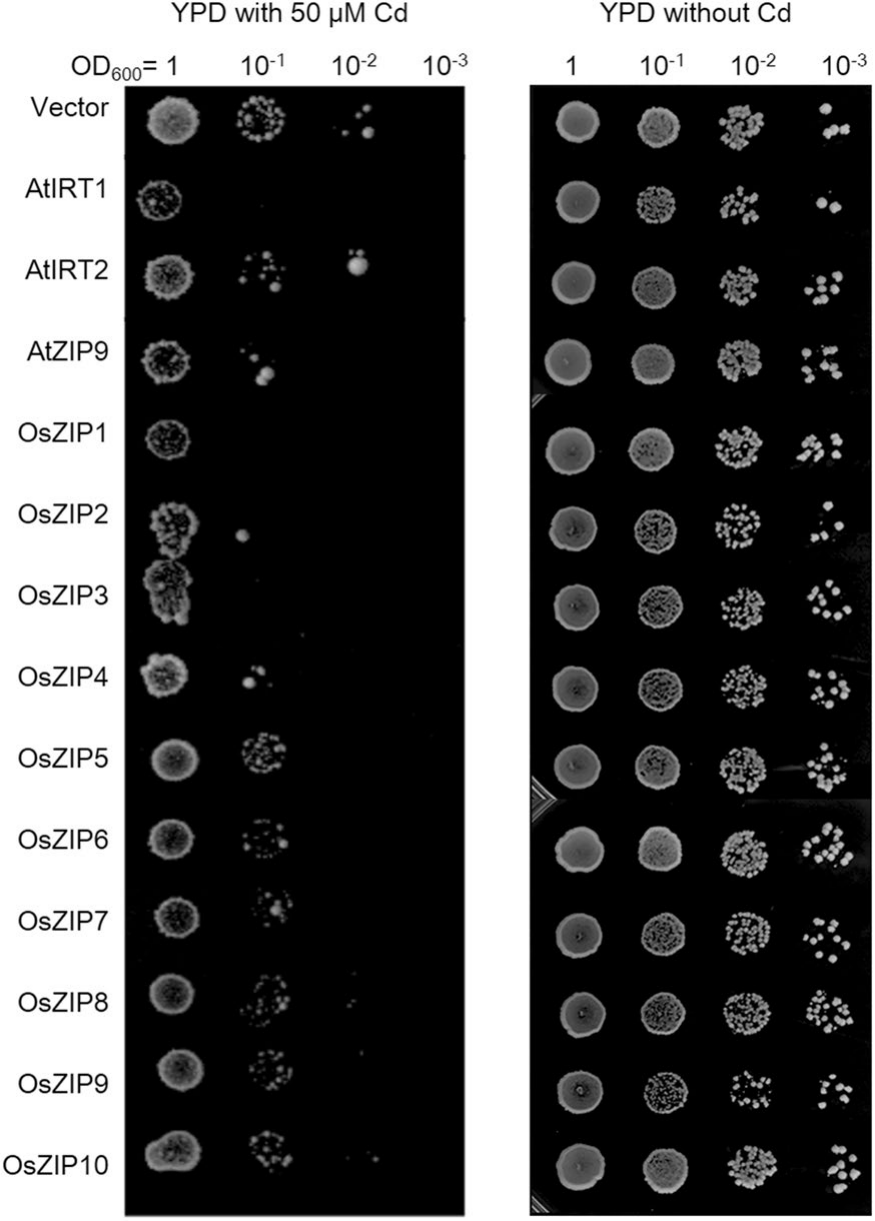
Drop assay for Cd sensitivity of yeast cells (*S. cerevisiae* AH109) expressing representative *ZIPs* tested in this study. The transformed cells expression *ZIPs* were subjected to a serial dilution (0–10^−4^) drop assay on YPD plates. 300 μg/mL G418 was added to maintain the vectors. Plates containing 50 μM CdCl_2_ were incubated at 28 °C for 5 days and growth state was subsequently observed. This experiment was performed three times

In *Arabidopsis*, Cd exposure impacted the expression of all ZIPs significantly. Strikingly, *AtIRT1* was induced with a 525-fold increase in shoot and a 22-fold increase in root (Fig. 2b). As abovementioned, some evidence already pointed to the Cd transport role of *AtIRT1* in yeast cells (Korshunova et al. 1999; Rogers et al. 2000; Vert et al. 2001; Eide et al. 1996) and in *Arabidopsis* (Fan et al. 2014; Connolly et al. 2002; Vert et al. 2002). Considering that *AtIRT1* is mainly expressed in root (Vert et al. 2002), AtIRT1 may function as a pump absorbing Cd from soil into root under sever Cd stress. A sharp increase of *AtIRT1* expression in shoot was also observed, indicating its potential role in Cd transport in shoot. Indeed, overexpression of *AtIRT1* in yeast increased the hosts’ sensitivity substantially (Fig. 3). Cd accumulation of yeast cells expressing *AtIRT1* was also increased by 40.1%, compared with the control (Additional file 1: Figure S4). Taken together, the results here further confirmed the role of *AtIRT1* in plant Cd uptake implied in previous studies (Rogers et al. 2000).

Like *AtIRT1*, *AtIRT2* was induced with a 1452-fold increase in shoot and a fourfold increase in root (Fig. 2b). Previous studies showed that *AtIRT2* overexpression increased Cd uptake of transgenic *Arabidopsis*, probably through the induction of *AtIRT1* expression (Vert et al. 2001, 2009). In this study, while both *AtIRT2* and *AtIRT1* were coincidently sharply induced when subject to Cd stress, overexpression of *AtIRT2* caused no significant changes in neither Cd sensitivity nor Cd accumulation (Fig. 3 and Additional file 1: Figure S4). It is thus very likely that *AtIRT2* worked indirectly and synergistically with *AtIRT1* in response to the Cd stress.

It was also highlighted that the expression of *AtZIP9* was significantly increased by ninefolds in shoot and 57-folds in root after Cd stress (Fig. 2b). Till now no evidence showed any role of *AtZIP9* in Cd uptake. The strong induction by Cd stress may imply its role in Cd transport, and its overexpression in yeast cells moderately increased hosts’ sensitivity to Cd. Conversely, expression of *AtZIP9* did not increase the Cd accumulation of host cells (Additional file 1: Figure S4). As a hypothetic transmembrane ion transporter, *AtZIP9* might affect the growth of host cells by a Cd-independent way. In addition, *AtIRT3*, *AtZIP4*, *AtZIP5*, *AtZIP11* and *AtZIP12* showed converse expression regulation when subject to the tested Cd stress, and *AtZIP7* was reduced in shoot and was under the detection limit in root (Fig. 2b). Their potential roles in Cd transport merit a further investigation.

In rice, homologous ZIPs responded differently from *Arabidopsis* to the Cd stress. Unlike in *Arabidopsis*, Cd stress increased the expression of most rice ZIPs in shoot but not root. These results imply that all these Cd-induced ZIPs involve in plant response to Cd. Except for *OsIRT2*, all ZIPs were significantly induced in rice shoot (Fig. 2b). Like *AtIRT3*, expression changes of *OsIRT1* and *OsZIP1* were converse in shoot and root (Fig. 2b). The positive role of *OsIRT1* and *OsZIP1* was demonstrated in the response of yeast and/or plant to Cd stress (Nakanishi et al. 2006; Lee and An 2009; Ramesh et al. 2003). Rice over-expressing *OsIRT1* showed reduced plant height and increased Cd accumulation under 300 μM Cd stress (Lee and An 2009), and the growth of *OsZIP1*-expressing yeast cells was inhibited by 10 μM Cd stress. In this study, the expression regulation of *OsIRT1* and *OsZIP1* in response to Cd stress was contrary between root and shoot. Rice might have a feedback regulation of *OsIRT1* and *OsZIP1* in root to prevent increasing Cd uptake from soil.

Os*ZIP1*-*10* were subject to Cd sensitivity and Cd accumulation tests. The expression of *OsZIP1* and *OsZIP3* in yeast caused an increased Cd sensitivity and Cd accumulation (Fig. 3 and Additional file 1: Figure S4), suggesting their potential roles in Cd uptake. This result is different from those by Ramesh et al. (2003), where yeast ZHY3 strains were used and different culture medium was applied. It was also noticed that *OsZIP6* did not caused an obvious increasing in Cd sensitivity (Fig. 3). This is not consistence with previous report, in which *Xenopus laevis* oocytes was used to test the Cd sensitivity (Kavitha et al. 2015). Different host and micro-environment may cause the altered conformation and activity of tested proteins. Expression of *OsZIP5*-*10* failed to alter Cd sensitivity and Cd accumulation of host cells obviously, implying that these ZIPs probably did not uptake Cd individually. Considering that *AtIRT2* involves in indirect Cd uptake in *Arabidopsis*, these Cd-induced ZIPs may also play roles in Cd uptake or transport indirectly. Their potential roles under Cd stress need further investigation using transgenic plants.

Indeed, this study showed that many ZIPs were significantly induced by Cd stress even the growth of seedling was inhibited obviously, and some of them increased hosts’ Cd sensitivity or Cd accumulation. These results will help to elucidate the genetic basis for Cd accumulation via a ZIP-dependent pathway in plants. Further analysis using transgenic plants will clarify the biological function of these ZIPs in plant Cd uptake and transport.

## Conclusions

In conclusion, this study revealed a distinct pattern in ZIPs genes expression regulation in response to Cd stress between *Arabidopsis* and rice. *Arabidopsis* mainly up-regulated root ZIPs genes, while rice mainly up-regulated shoot ZIPs genes. Interestingly, some genes like *AtIRT3*, *AtZIP5*, *AtZIP12*, *OsIRT1* and *OsZIP1* showed contrary expression regulation when subject to the tested Cd stress. Three genes, *AtIRT1*, *OsZIP1* and *OsZIP3*, conferred an increased sensitivity to Cd stress and more Cd accumulation when expressed in yeast cells, implying a role in direct Cd uptake in plants.

## Additional file

**Additional file 1: Table S1.** The qPCR primers used in this study. **Table S2.** Primers used in plasmid construction. **Figure S1.** Genome locations of 27 ZIP genes in *Arabidopsis* (A) and rice (B). Information were acquired in the PLAZA database and plotted using Photoshop CS6. **Figure S2.** Evolutionary relationships of ZIP family genes and their structures. The Neighbor-Joining tree was produced using MEGA7 with 1,000 bootstrap replicates, and the gene structures was predicted using Gene Structure Display Server. Dark blue boxes indicate exons; black lines indicate introns; light blue boxes indicate untranslated regions. **Figure S3.** Effect of Cd stress on root length (A and B) and dry weight (C and D) of *Arabidopsis* and rice. (for root length, n=20; for dry weight, n=3. Student *t* test, * indicates P<0.05). **Figure S4.** Effect of *ZIPs* on Cd accumulation. Cells expressing *ZIPs* were incubated using liquid YPD medium plus 300 μg/mL G418 and 50 μM Cd for 12 h, after which the Cd concentration of each strain was measured by an atomic absorption spectrometer method. Cells harboring empty pCEV-G1-Km (Vector) was used as a negative control. (n=3, student *t* test, * P < 0.05).

## Abbreviations

ZIPs: zinc(Zn)-regulated/iron(Fe)-regulated transporter-like family proteins
Cd: cadmium
TM: transmembrane
Zn: zinc
Fe: iron
Mn: manganese
Cu: copper
