# Supplementary material for: Arabidopsis and rice showed a distinct pattern in ZIPs genes expression profile in response to Cd stress
Source: Bot Stud. 2018 Sep 25;59:22. doi: 10.1186/s40529-018-0238-6 (PMC6156699; doi:10.1186/s40529-018-0238-6)
Supplement: Supplementary file 1 — Additional file 1: Table S1. The qPCR primers used in this study. Table S2. Primers used in plasmid construction. Figure S1. Genome locations of 27 ZIP genes in Arabidopsis (A) and rice (B). Information were acquired in the PLAZA database and plotted using Photoshop CS6. Figure S2. Evolutionary relationships of ZIP family genes and their structures. The Neighbor-Joining tree was produced using MEGA7 with 1,000 bootstrap replicates, and the gene structures was predicted using Gene Structure Display Server. Dark blue boxes indicate exons; black lines indicate introns; light blue boxes indicate untranslated regions. Figure S3. Effect of Cd stress on root length (A and B) and dry weight (C and D) of Arabidopsis and rice. (for root length, n=20; for dry weight, n=3. Student t test, * indicates P<0.05). Figure S4. Effect of ZIPs on Cd accumulation. Cells expressing ZIPs were incubated using liquid YPD medium plus 300 μg/mL G418 and 50 μM Cd for 12 h, after which the Cd concentration of each strain was measured by an atomic absorption spectrometer method. Cells harboring empty pCEV-G1-Km (Vector) was used as a negative control. (n=3, student t test, * P < 0.05). [file 40529_2018_238_MOESM1_ESM.docx]

**Additional information for**

***Arabidopsis* and rice** **showed a distinct pattern in ZIPs genes expression profile in response to Cd stress**

Xin Zheng^a^, Liang Chen^a^, Xiaofang Li^a,b*^

^a^ *Key Laboratory for Agricultural Water Resources, Center for Agricultural Resources Research, Institute of Genetics and Developmental Biology, Chinese Academy of Sciences, Shijiazhuang, Hebei 050021, P. R. China*

^b^ *Environment Center, Sustainable Minerals Institute, The University of Queensland, Brisbane, Queensland 4072, Australia*

^*^Correspondence

Xiaofang Li (xfli@sjziam.ac.cn; x.li10@uq.edu.au)

Table S1. The qPCR primers used in this study.

| **Gene** | **Forward primer** | **Reverse primer** |
| --- | --- | --- |
| AtIRT1 | TTGCCATGGTCTTGGAACTTG | CCGAATGGTGTTGTTACCGC |
| AtZIP8 | GTTGCATCCTTCAGCGTTTACA | GGACTGACATTCCGCCACA |
| AtIRT2 | TGCATCCTCCAGGCAGATTT | GACCCCATGAACTCGGTAGC |
| AtZIP10 | CATCCTTCAGGCGGAGTACG | TCGGCAGCTAGAAGGTCAAC |
| AtZIP5 | TTGGCGTGGAATCTGGTGAA | TTGAAATTTCCCTGGGCGATG |
| AtZIP3 | TGCACAGGTATTGGAGTTGGG | TGCGAAGAACGTCGACATGA |
| AtZIP1 | TGTCGCAGGTGCTTGAGATT | CATATCCGCCAGGGAGATGC |
| AtZIP12 | GATCCAAAGGTGGCTCTGCT | GGTTCTTCTCCAAGGCACGA |
| AtZIP7 | GGTTCCATCTTACAAGCGCAAT | CAACAATCCTGCAGAGCACG |
| AtZIP6 | TGCTCAGGCTGGGTTTAAGG | CGCAAGTCGTCAGCATCTTG |
| AtZIP9 | ACTCTGCTCGCGGATTTCAT | TGGAGAGGCTATGACGGTGA |
| AtZIP4 | CACGGACATATGCACGGGAA | ACTGTGCCTGAGAGATGCAG |
| AtIRT3 | ATGGACACGGACATGGACAC | CTGCGATTAGAGGCCGGATT |
| AtZIP11 | TCCGTATGATCCCTGACCGT | ACAAATACCCCACACGCCAA |
| AtZIP2 | GAAGAGGAGACCAACCAGCC | TCCGAGGAGGAGAAACGACT |
| AtUBC | CTGCGACTCAGGGAATCTTCTAA | TTGTGCCATTGAATTGAACCC |
| OsIRT1 | CCGCGTCGTCGTTCAGG | GAAGAAGACGAGCACCGACC |
| OsIRT2 | TCATGCTCACGTTCCACACG | TGCACAATGACGCGATTCCT |
| OsZIP1 | GGGTTTCTTCGCTCCTTGTCG | AGGAGGAAGCTCTCGTTCCA |
| OsZIP2 | ACAAATGGCAACGCCTCTGA | CATGCGTCAGCCTTCGTCTC |
| OsZIP3 | GGTGGTGGACTCATTGGCTG | TGAACCCTCCTCTGGTGAGC |
| OsZIP6 | GTTCGAAGGAATGGGCCTCG | GAAAGTGAGCCAAGGAGCCC |
| OsZIP7 | GGCGTCATTCTACAACGCCA | GGCCCAGATTGCAAGGGATG |
| OsZIP9 | GTGCATCCGAGAGCCCAAG | GCCGTGGGGCTATTCTCATTG |
| OsZIP5 | CCGTGTCAGGTCAGTGGTGA | GCGCCATGTAGACGAGTATCC |
| OsZIP10 | CGTCTCTCAGAGCCCGTGTA | ACAGTGATCCCAGCAGGTGT |
| OsZIP8 | GGCATCGTGTCATCTCTCAGG | GACCTCACCTTGAACTTCGCC |
| OsZIP4 | GTCGTCAATCAGGCCACTCG | GAGATTGCGATCCCCAGTGC |
| OsActin1 | AGACCTTCAACACCCCTGCT | TAACCACGCTCCGTCAGGAT |

Table S2. Primers used in plasmid construction.

| **Gene** | **Forward primer** | **Reverse primer** |
| --- | --- | --- |
| AtIRT1 | cgcggatccATGGCTTCAAATTCAGCACTT | cggggtaccTTAAGCCCATTTGGCGATAAT |
| AtIRT2 | cgcggatccATGGCTACTACCAAGCTCGTCTAC | cggggtaccTTAAGCCCACACGGCGAC |
| AtZIP9 | tcccccgggATGGCGTCGATCCTTATCTC | cggggtaccTCAAGCCCAAATTGCAAGAGC |
| OsZIP1 | cgcggatccATGGCCAGGACGATGACGATG | cggggtaccTCAGTCCCAGATCATGACGACAGCCA |
| OsZIP2 | cgcggatccATGGCGGGAGGCAGG | cggggtaccTCAGGTGTCCCAGATCATGACGA |
| OsZIP3 | cgcggatccATGGGAGCCAAGAAGCATAC | cggggtaccCTATGCCCATATGGCAAGCA |
| OsZIP4 | cgcggatccATGGACGCCATGAGGCA | cggggtaccTCATGCCCATATGGCAAGCA |
| OsZIP5 | cgcggatccATGGCGACGGCGGCG | cggggtaccTCACGCCCAGATGGCGATCAT |
| OsZIP6 | cgcggatccATGTCGGGGACGGGGTGC | cggggtaccCTATGCCCAGAGAGCTAATATAGACATG |
| OsZIP7 | cgcggatccATGGAGCGGTTCGTGCAGTTC | cggggtaccTCAGGCCCAGATTGCAAGGGAT |
| OsZIP8 | tcccccgggATGAGGACGAACACCACCGC | cggggtaccCTAGGCCCATTTGGCGAGCA |
| OsZIP9 | cgcggatccATGGCTTTCGATCTCAAGCT | cggggtaccTCAAGCCCAAATACCAAGCAA |
| OsZIP10 | cgcggatccATGGAGTCGTCGTCGTCG | cggggtaccCTAAGCCCATAATGCCAGG |

Figure S1


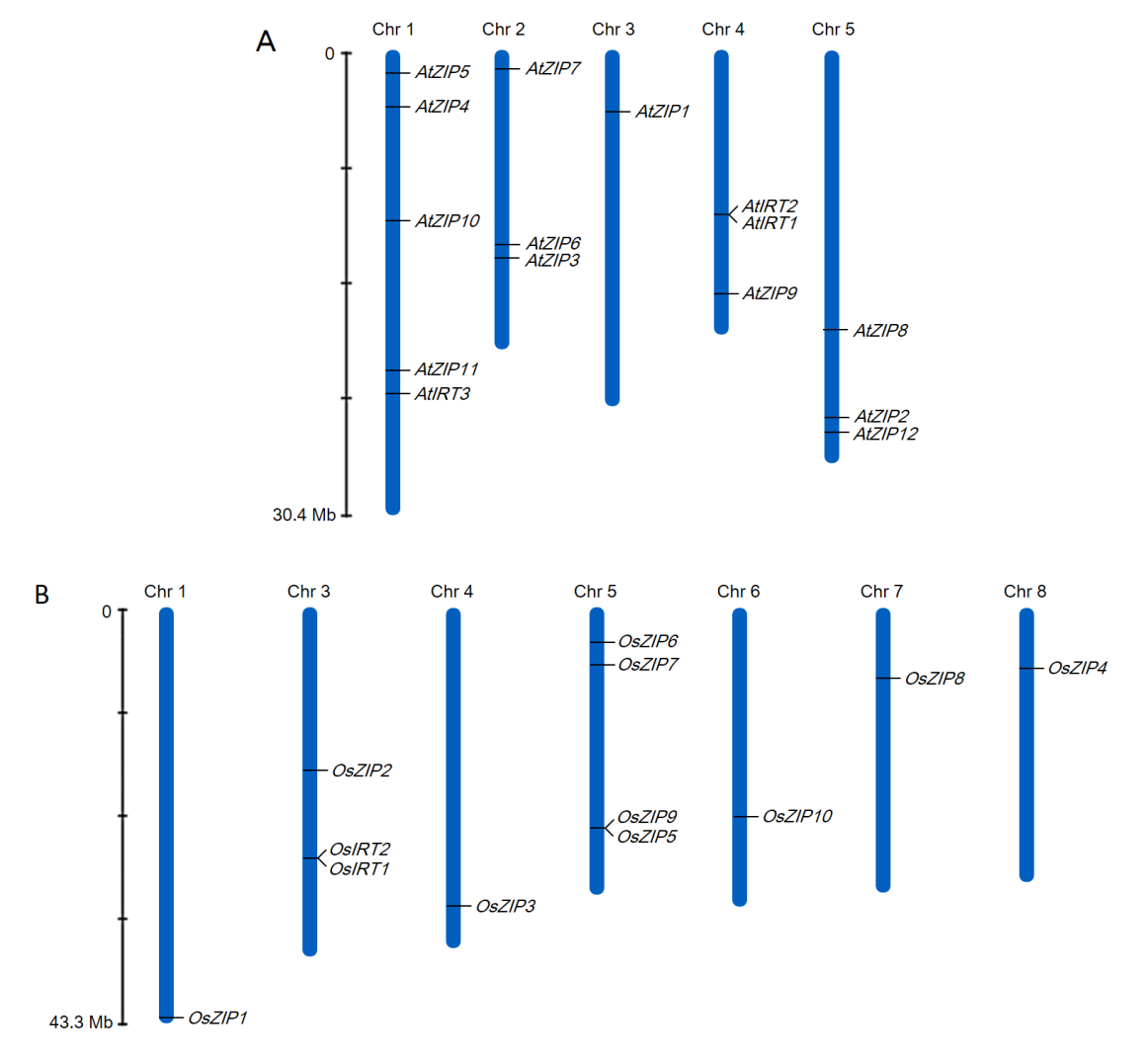


Figure S1. Genome locations of 27 ZIP genes in *Arabidopsis* (A) and rice (B). Information were acquired in the PLAZA database and plotted using Photoshop CS6.

Figure S2


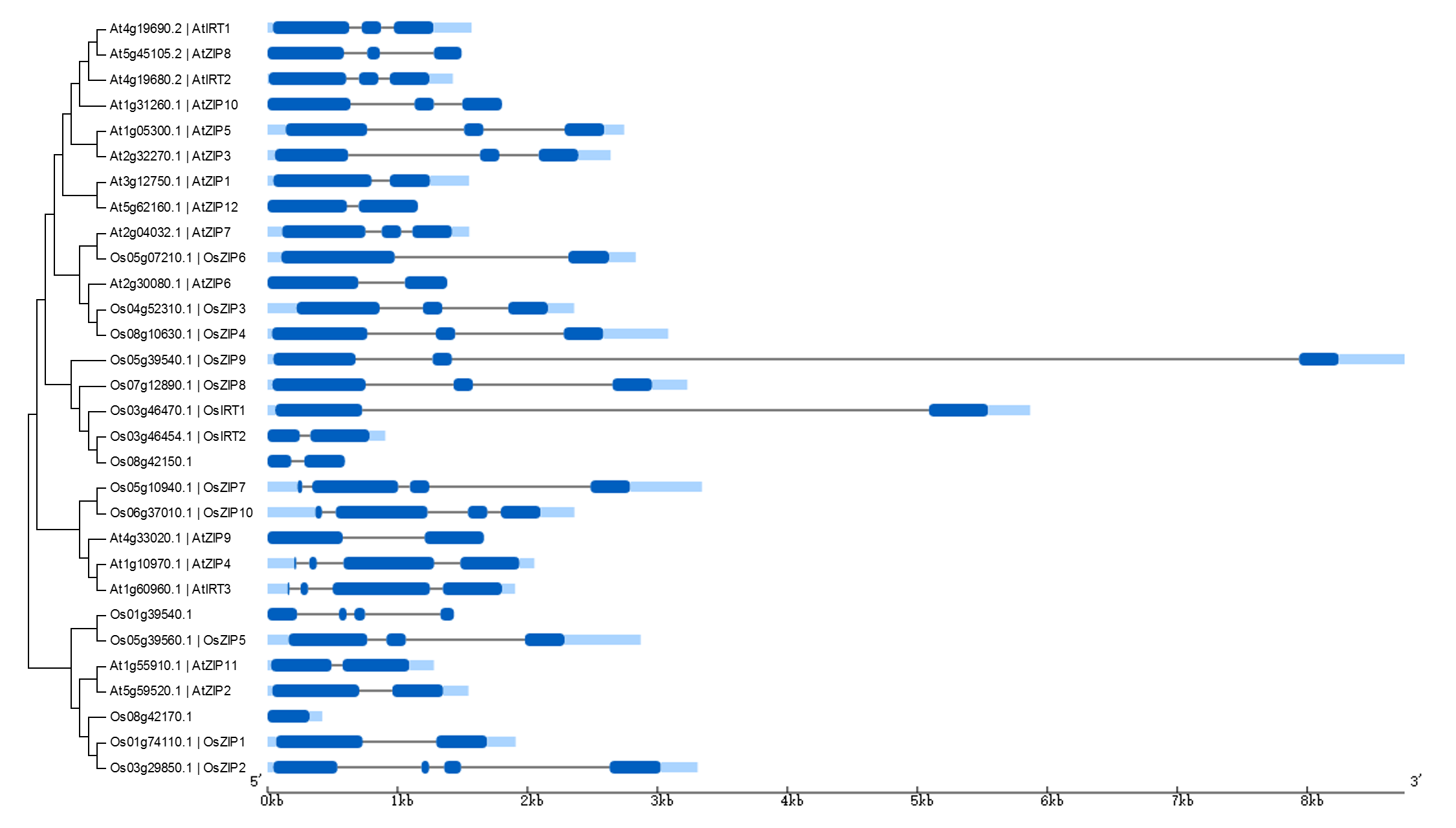


Figure S2. Evolutionary relationships of ZIP family genes and their structures. The Neighbor-Joining tree was produced using MEGA7 with 1,000 bootstrap replicates, and the gene structures was predicted using Gene Structure Display Server. Dark blue boxes indicate exons; black lines indicate introns; light blue boxes indicate untranslated regions.

Figure S3


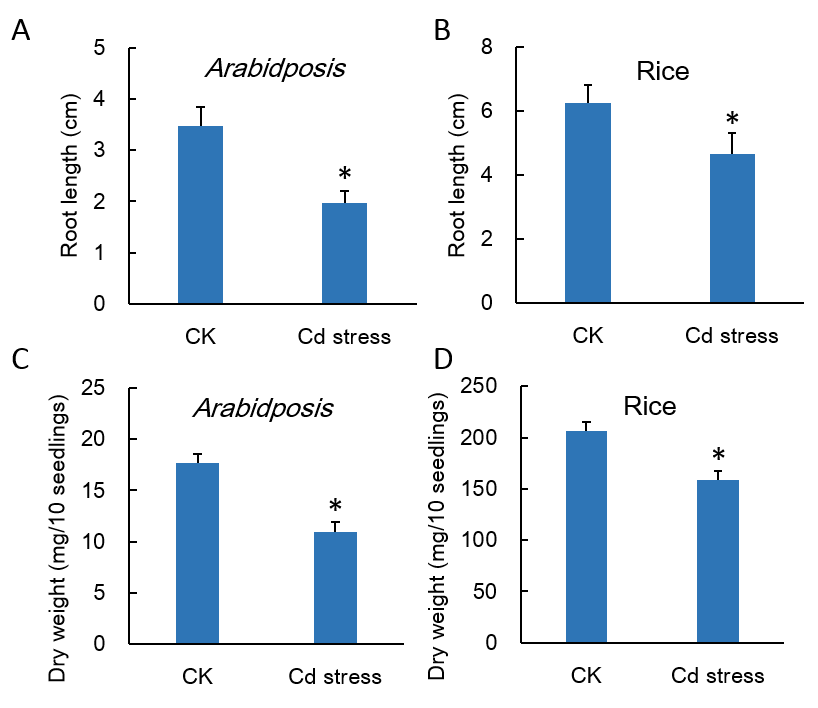


Figure S3. Effect of Cd stress on root length (A and B) and dry weight (C and D) of *Arabidopsis* and rice. (for root length, n=20; for dry weight, n=3. Student *t* test, * indicates P<0.05).

Figure S4


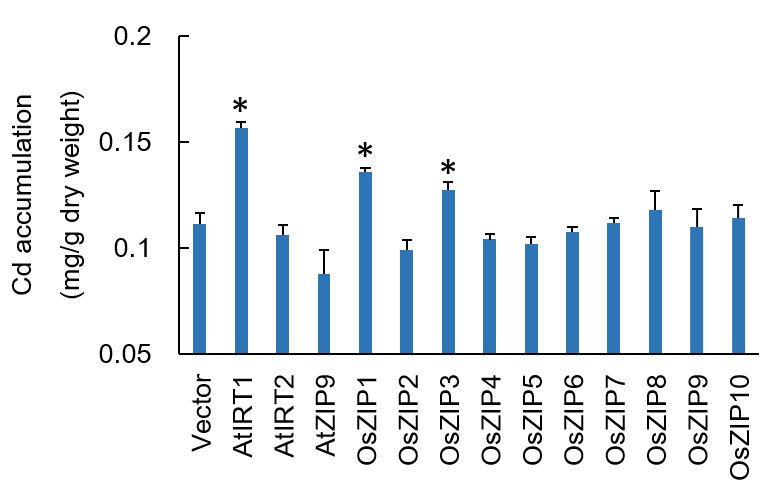


Figure S4. Effect of *ZIPs* on Cd accumulation. Cells expressing *ZIPs* were incubated using liquid YPD medium plus 300 μg/mL G418 and 50 μM Cd for 12 h, after which the Cd concentration of each strain was measured by an atomic absorption spectrometer method. Cells harboring empty pCEV-G1-Km (Vector) was used as a negative control. (n=3, student *t* test, * P < 0.05)
